# Evaluation of Lung Aeration and Respiratory System Mechanics in Obese Dogs Ventilated With Tidal Volumes Based on Ideal vs. Current Body Weight

**DOI:** 10.3389/fvets.2021.704863

**Published:** 2021-10-01

**Authors:** Joaquin Araos, Luca Lacitignola, Valentina de Monte, Marzia Stabile, Ian Porter, Daniel E. Hurtado, Agustín Perez, Antonio Crovace, Salvatore Grasso, Manuel Martin-Flores, Francesco Staffieri

**Affiliations:** ^1^Department of Clinical Sciences, College of Veterinary Medicine, Cornell University, Ithaca, NY, United States; ^2^Section of Veterinary Clinics and Animal Production, Department of Emergency and Organ Transplantation D.E.O.T., “Aldo Moro” University of Bari, Bari, Italy; ^3^Department of Structural and Geotechnical Engineering, School of Engineering, Pontificia Universidad Catolica de Chile, Santiago, Chile; ^4^Institute for Biological and Medical Engineering, Schools of Engineering, Medicine and Biological Sciences, Pontificia Universidad Catolica de Chile, Santiago, Chile; ^5^Millennium Nucleus for Cardiovascular Magnetic Resonance, Santiago, Chile; ^6^Section of Anesthesia and Intensive Care, Department of Emergency and Organ Transplantation D.E.O.T., “Aldo Moro” University of Bari, Bari, Italy

**Keywords:** obese, dogs, mechanical ventilation, ideal body weight, current body weight, anesthesia

## Abstract

We describe the respiratory mechanics and lung aeration in anesthetized obese dogs ventilated with tidal volumes (VT) based on ideal (VTi) vs. current (VTc) body weight. Six dogs with body condition scores ≥ 8/9 were included. End-expiratory respiratory mechanics and end-expiratory CT-scan were obtained at baseline for each dog. Thereafter, dogs were ventilated with VT 15 ml kg^−1^ based on VTi and VTc, applied randomly. Respiratory mechanics and CT-scan were repeated at end-inspiration during VTi and VTc. Data analyzed with linear mixed models and reported as mean ± SD or median [range]. Statistical significance *p* < 0.05. The elastance of the lung, chest wall and respiratory system indexed by ideal body weight (IBW) were positively correlated with body fat percentage, whereas the functional residual capacity indexed by IBW was negatively correlated with body fat percentage. At end-expiration, aeration (%) was: hyperaeration 0.03 [0.00–3.35], normoaeration 69.7 [44.6–82.2], hypoaeration 29.3 [13.6–49.4] and nonaeration (1.06% [0.37–6.02]). Next to the diaphragm, normoaeration dropped to 12 ± 11% and hypoaeration increased to 90 ± 8%. No differences in aeration between groups were found at end-inspiration. Airway driving pressure (cm H_2_O) was higher (*p* = 0.002) during VTc (9.8 ± 0.7) compared with VTi (7.6 ± 0.4). Lung strain was higher (*p* = 0.014) during VTc (55 ± 21%) than VTi (38 ± 10%). The stress index was higher (*p* = 0.012) during VTc (SI = 1.07 [0.14]) compared with VTi (SI = 0.93 [0.18]). This study indicates that body fat percentage influences the magnitude of lung, chest wall, and total respiratory system elastance and resistance, as well as functional residual capacity. Further, these results indicate that obese dogs have extensive areas of hypoaerated lungs, especially in caudodorsal regions. Finally, lung strain and airway driving pressure, surrogates of lung deformation, are higher during VTc than during VTi, suggesting that in obese anesthetized dogs, ventilation protocols based on IBW may be advantageous.

## Introduction

Overweight and obesity, defined as the excessive accumulation of adipose tissue, is becoming more common in veterinary patients, with studies reporting the prevalence of combined overweight and obesity as high as 45% (1).

In people, increased deposits of abdominal and intrathoracic fat result in significant alterations in respiratory mechanics, lung volumes, and gas exchange (2). In humans undergoing general anesthesia, functional residual capacity (FRC) decreases exponentially as body mass index increases (2). Although a decrease in FRC is also described for lean anesthetized people, the increased abdominal content and intraabdominal pressure result in increased loads on certain lung regions in the obese. Increased respiratory system elastance—the reciprocal of compliance-, impaired gas exchange and increased respiratory system resistance (R_aw_) usually accompany the reductions in FRC. Therefore, mechanical ventilation in obese human patients is challenging, and requires careful planning to prevent the development of postoperative pulmonary complications (3). For instance, tidal volume (VT) in obese human patients undergoing mechanical ventilation is calculated based on predicted, instead of current, body weight (3). In obese mice, mechanical ventilation using VT based on current body weight results in altered lung mechanics and associated lung inflammation, when compared with ventilation using ideal body weight (IBW) (4).

Obese dogs share many similarities with obese people (5). For instance, it has been shown that awake obese dogs have reduced FRC and VT and increased respiratory rates (6–8). Sedated obese dogs have been shown to have impaired oxygenation (7). There is, however, very little information regarding the respiratory mechanics and lung aeration in obese dogs undergoing general anesthesia and mechanical ventilation, and most of the available information is extrapolated from obese people and experimental research. Moreover, it is unknown whether in obese dogs, VT should be calculated based on ideal vs. current body weight.

In the present study, we aimed at characterizing the respiratory system mechanics, gas exchange, and lung aeration in a small cohort of anesthetized and mechanically ventilated obese dogs. Moreover, we aimed at comparing the effects of using a VT calculated based on current vs. ideal body weight. We hypothesized that this population of obese dogs would have large areas of hypo or nonaerated lung tissue, especially in the caudodorsal regions, and that a ventilation protocol using VT based on ideal, as compared to current body weight, would result in decreased airway pressures and improved lung aeration.

## Materials and Methods

This study was approved by the Ethical Committee for Clinical Study in Animal Patients of the Department of Emergency and Organ Transplantation, University of Bari, Italy (Protocol 11/2017).

### Animals

This was a prospective, randomized, controlled clinical trial that included client-owned dogs presented to the Veterinary Hospital at the University of Bari. For each case, a written consent form was signed by the dog owners, in compliance with the Italian Welfare Act and Statutes of the University of Bari. The study population consisted of dogs of different breeds referred to the Section of Veterinary Clinics and Animal Production of the Department of Emergency and Organ Transplantation (DEOT). All dogs were affected by abdominal or mammary gland tumors and were scheduled for a thoracic CT scan to evaluate for the presence of pulmonary metastasis. All patients underwent a complete physical examination and a standard radiographic examination of the thorax before induction of general anesthesia. Body condition score (BCS) was assessed based on a 9 point validated scale considering clinical inspection and body palpation (9). Although, there is not an established criterion in veterinary medicine, for the purpose of this study, only dogs with a BCS of ≥ 8 out of 9 were considered obese and were included in the study (10). Therefore, the inclusion criteria for the study was a BCS ≥ 8 out of 9, a normal chest radiograph and the absence of cardiac disease at a stage ≥ than B2, based on the ACVIM classification. Dogs in which pulmonary metastasis or other major lung pathologies were detected in the CT scans were excluded from the study. Abnormal blood tests results were not considered as criteria for exclusion from the study.

Dogs were also classified as barrel-chested, deep-chested or intermediate-chested (11).

### Estimation of Ideal Body Weight

Current body weight was determined before the induction of anesthesia using an electronic scale. Ideal body weight was calculated from the current body weight based on the following formula:


$$\begin{array}{l} \text{Ideal body weight }(\text{kg})\;=\text{current body weight }-\;[\text{body fat }(\%)\\ \;\times \text{ current body weight}] \end{array}$$

Where body fat (%) corresponds to the percentage of body fat computed based on the following morphometric gender specific formulas (12):


$$\begin{array}{l} \text{ Males }\left(\text{\% body fat}\right)\;=\;-1.4\;\left(\text{HS cm}\right)\;+\;0.77\;\left(\text{PC cm}\right)\;+\;4\\ \text{Females }\left(\text{\% body fat}\right)\;=\;-1.7\;\left(\text{HS cm}\right)\;+\;0.93\;\left(\text{PC cm}\right)\;+\;5 \end{array}$$

Where HS correspond to the length (in cm) from tuber calcaneus to midpatellar ligament (i.e., hock-to-stifle length) and PC to the pelvic circumference measured around the level of the flank.

### Anesthesia and Monitoring

All dogs were sedated with acepromazine (Prequillan 1%, Fatro Spa, 20 μg kg^−1^) intramuscularly (IM) followed 20 min later with IM morphine (Morfina Cloridrato 1%, Molteni Spa, 0.3 mg kg^−1^). General anesthesia was induced with intravenous propofol titrated to effect (Proposure 1%, Merial Spa, 5–7 mg kg^−1^). After orotracheal intubation with a cuffed endotracheal tube (Teleflex Medical Srl, Italy), dogs were positioned in dorsal recumbency and anesthesia was maintained with isoflurane (IsoFlo, Virbac Spa, Italy) in oxygen. Dogs were then connected to a mechanical ventilator (Siemens Servo 900C, Siemens Elma) equipped with a dedicated vaporizer (Siemens 950, Siemens Elma) and a standard nonrebreathing circuit (Flextube; Intersurgical Ltd, Italy). Dogs were ventilated in the volume-controlled mode with a VT adjusted according to the study protocol (see below), inspiratory to expiratory time ratio 1:2, an inspiratory pause 25% of the inspiratory time and the respiratory rate adjusted to maintain the end-tidal partial pressure of carbon dioxide between 35 and 45 mmHg. The end-tidal concentration of isoflurane was maintained between 1.3 and 1.5%. A lactated Ringer solution was infused at 5 ml kg h^−1^ during anesthesia. Pulse oximetry, heart rate and rhythm, esophageal temperature, end-tidal CO_2_ and noninvasive arterial blood pressure were continuously monitored with a multiparametrer monitor (SC 6002XL, Siemens, MA).

### Study Protocol

**Figure 1:**
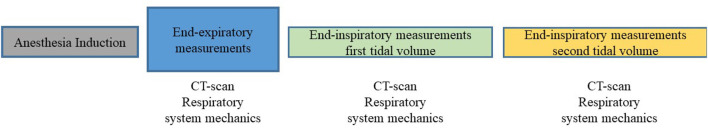
Flowchart illustrating the experimental design. After induction of anesthesia, six obese dogs undergoing computed tomography (CT) scans were ventilated with a tidal volume of 15 ml kg^−1^ of current (VTc) and ideal (VTi) body weight, applied in random order. On each experiment, end-expiratory CT scans and respiratory system mechanics were recorded once. At the end of each study treatment (VTc and VTi) end-inspiratory CT images, respiratory system mechanics and arterial blood gases were obtained.

During volume-controlled ventilation, dogs were ventilated with a VT of 15 ml kg^−1^ based on the current or IBW. Each dog received, in the same anesthetic episode, 20 min of ventilation with VT based on the IBW (VTi) and 20 min of ventilation based on the current body weight (VTc). The sequence of the treatments was assigned based on a simple randomization protocol, which relied on a predetermined table. The sequence of the treatments was based on a simple randomization protocol (MedCalc Software version 19.7.4, Belgium) which established the assignment of each dog to group A or group B. Group A received first the VTi and then the VTc ventilation protocol. The sequence of the events in the Group B was the reversed. On each experiment, end-expiratory CT images of the thorax and respiratory system mechanics were obtained once, 15 min after anesthesia induction. At the end of each study treatment (VTc and VTi) end-inspiratory CT images of the thorax, respiratory system mechanics and arterial blood gases were obtained. Therefore, for each dog, we report 1 set of baseline end-expiratory images and 2 sets of end-inspiratory images, respiratory mechanics, and arterial blood gases (see Figure 1). Arterial blood samples were collected before measurements of respiratory mechanics and lung aeration and analyzed immediately (Idexx VetStat, Idexx Laboratories Inc., ME). Measured blood gases were corrected for the esophageal temperature of the dog (maintained between 37 and 38.5°C throughout the procedure) at the time of sample collection. Computed tomography images were obtained during muscle relaxation, achieved with an intravenous bolus (0.4 mg kg^−1^) of rocuronium (Rocuronium Kabi 10 mg mL^−1^, Italy). At the end of the study, the train-of-four (TOF) ratio was measured over the peroneal nerve (TOF-Watch, Organon, Dublin, Ireland) and a dose of neostigmine (0.02 mg kg^−1^, Intrastigmina, Italy) was given immediately after a dose of atropine (0.02 mg/kg, Atropina Solfato, Ati Srl, Italy) if the TOF activity was < 0.9 (13). Dogs were discharged from the hospital at the end of the day.

The oxygen content-based index F-shunt was calculated using validated formulas (14).

### CT Scan

#### Lung Volumes and Aeration Distribution

The methodology for CT scanning and calculation of lung aeration has been described elsewhere (15). Briefly, full thoracic CT scans were performed (GE ProSpeed sx, General Electric, New York, NY). End-expiratory images were obtained 15 min after anesthesia induction at an airway pressure (P_aw_) of zero cmH_2_O, by disconnecting the patient from the circuit and clamping the endotracheal tube. End-inspiratory images were taken at the end of a 4-s inspiratory pause. Pressure was maintained constant inside the lung by clamping the endotracheal tube during the pauses. The clamp was then released and baseline ventilator settings resumed. End-inspiratory images were obtained at the end of a 20-min period of ventilation with both VTc and VTi.

CT images were obtained at 120 kVp and 160 mA; the matrix size was 512 × 512, field of view 35 cm, and pitch 1.5. Images were reconstructed with 10 mm slice thickness and analyzed with the VELAS software® (Veterinary version of Maluna® by P. Herrmann, University of Göttingen Germany). Both right and left lungs were analyzed. The outer boundary of the lung was traced along the inner aspect of the ribs and the inner boundary along the mediastinal organs. Areas of the hilum containing the trachea, main bronchi, and vessels were excluded. Functional residual capacity (mL) and end-inspiratory lung volumes (EILV, mL) were computed by including pixels with densities between −1.000 and 100 HUs (CT number) during the end-expiratory and end-inspiratory pauses, respectively. Volumes were calculated according to the following formula:


$$\begin{array}{l} \text{ Gas volume }\left(\text{mL}\right)\;=\text{ CT number}/\left(-1.000\right)\;\times \text{ voxel volume} \end{array}$$

Where each voxel is a pixel with a square base of 0.59 mm on each side and a height corresponding to the CT slice thickness (10 mm). For each dog, the data acquired in each CT image were then added together to yield the total lung volumes at end-expiration and end-inspiration. Lung strain was calculated as:


$$\begin{array}{l} \text{ Global lung strain }\left(\text{\%}\right)\;=\;\left[\left(\text{EILV }-\text{ FRC}\right)/\text{FRC}\right]\;\times \;100 \end{array}$$

For the analysis of global lung aeration, lung tissue was classified according to its gas/tissue content as hyperaerated (−1,000 to −901 HUs), normally aerated (−900 to −501 HUs), hypoaerated (−500 to −101 HUs), or nonaerated (−100 to 100 HUs) (15). To evaluate differences in aeration in the ventrodorsal direction, the software semi-automatically divided the lung into three equal regions of interest (ventral, intermediate, and dorsal). Aeration was also evaluated in the craniocaudal direction by plotting the calculated aeration for each axial slide.

In addition, the end-expiratory CT scan of an obese dog from this study and an end-expiratory CT scan from a lean dog of an unrelated, ongoing study, were used to create a 3D reconstruction of the lungs showing only the hypoaerated tissue. The DICOM data of each dog was imported into specialized segmentation software (Mimics Research, version 21, Leuven, Belgium) and thresholding masks were applied to segments of hypoaerated lung tissue. The mask for this lung category was manually split to remove any regions outside of the lung which may have been included based on the threshold values. The resulting models were exported in STL format, converted to OBJ format, combined into a single file using Blender (www.blender.org) and uploaded to Sketchfab (www.sketchfab.com) for display. A MP4 video was created from the Sketchfab model using the screen capture feature in PowerPoint (Microsoft, USA).

### Regional Volumetric Lung Strain in One Dog

Regional volumetric strain in the lung parenchyma of one dog was determined from CT lung images following the biomechanical analysis method developed by Hurtado et al. (16, 17). A strain analysis was performed based on a tetrahedral mesh of the lung reconstructed from a segmentation of the end-expiratory image. To report the results, regions of interest (ROIs) were defined by grouping sets of neighboring elements of the lung mesh so that ROIs divided the domain along the craniocaudal and ventrodorsal directions in roughly equal volume compartments. Both directions were intersected to allow for a two-dimensional spatial analysis of the quantities of interest. These directions, when intersected, gave a matrix of 5 × 5 ROIs, each with independent information on regional volumetric strain. Where the craniocaudal and dorsoventral segments did not intersect, that region was void. This technique is currently very time consuming and expensive, explaining the reason to include only one dog that best represented the average observed results.

### Respiratory System Mechanics

The methodology for evaluation of respiratory mechanics has been described elsewhere (15). Briefly, gas flow was measured with a calibrated heated pneumotachograph (Fleisch No. 2, Fleisch, Switzerland) connected to a calibrated differential pressure transducer (Diff-Cap, Special Instruments GmbH, Germany). Prior to each experiment, it was verified that the pneumotachogram was linear over the range of gas flows. Tidal volume was obtained by integration of the flow signal. The P_aw_ (mmHg) was measured proximal to the endotracheal tube.

A 5-Fr balloon-tipped catheter system (Cooper Surgical®, USA) was used to measure esophageal pressure, as an estimate of pleural pressure. An esophageal balloon (volume, 10 mL) was introduced through the dog's mouth and advanced within the distal third of the esophagus. The catheter then connected to a pressure transducer. The balloon was then filled with ~1 mL of air, following the description by Mauri et al. (18). Briefly, the most adequate filling volume of the balloon was determined by progressively inflating the balloon, within the catheter specific range of filling volumes, finally selecting the lowest volume associated with the largest tidal swings. Finally, correct catheter position was confirmed by observation of cardiac oscillations and by performance of the positive pressure occlusion technique (18). Variables were displayed and collected for posterior analysis through a 12-bit analog-to-digital converter board (DAQCard 700; National Instruments, TX, USA) at a sampling rate of 200 Hz (ICU Lab, KleisTEK Engineering, Italy).

The static mechanical properties were determined by applying 4-s end-inspiratory and end-expiratory airway occlusions at the time of end-inspiratory and end-expiratory CT imaging, respectively (19). Total end-expiratory pressure of the respiratory system (PEEP_RS_) and of the chest wall (PEEP_CW_) were measured as the plateau pressure in P_aw_ and P_ES_, respectively, at the end of the expiratory pause.

The airway driving pressure (ΔP_awO_) and the tidal variation in esophageal pressure (ΔP_ES_) (20) were calculated as:


$$\begin{array}{l} \;\Delta {\text{P}}_{\text{awO}}\;={\text{P}}_{\text{plat}}\;-\text{PEE}{\text{P}}_{\text{RS}}\\ \;\Delta {\text{P}}_{\text{ES}}\;={\text{P}}_{\text{ESplat}}\;-\text{PEE}{\text{P}}_{\text{CW}} \end{array}$$

Where P_plat_ is the plateau pressure measured at the end of the 4-s inspiratory pause. Thereafter, the transpulmonary driving pressure (ΔP_L_) was calculated as:


$$\begin{array}{l} \;\Delta {\text{P}}_{\text{L}}\;=\;\Delta {\text{P}}_{\text{awO}}\;-\;\Delta {\text{P}}_{\text{ES}} \end{array}$$

Since in humans obesity has been shown to induce negative end-expiratory P_L_, this variable was also calculated as:


$$\begin{array}{l} \text{ End}-\text{expiratory }{\text{P}}_{\text{L}}\;=\text{ PEE}{\text{P}}_{\text{RS}}\;-\text{PEE}{\text{P}}_{\text{CW}} \end{array}$$

The elastance of the respiratory system (E_RS_), lung (E_L_), and chest wall (E_CW_) were calculated as:


$$\begin{array}{l} \;{\text{E}}_{\text{RS}}\;\left(\text{cm}{\text{H}}_{2}\text{O m}{\text{L}}^{-1}\right)\;=\Delta {\text{P}}_{\text{awO}}/\text{VT}\\ \;{\text{E}}_{\text{L}}\;\left(\text{cm}{\text{H}}_{2}\text{O m}{\text{L}}^{-1}\right)\;=\Delta {\text{P}}_{\text{L}}/\text{VT}\\ \;{\text{E}}_{\text{CW}}\;\left(\text{cm}{\text{H}}_{2}\text{O m}{\text{L}}^{-1}\right)\;=\Delta {\text{P}}_{\text{ES}}/\text{VT} \end{array}$$

Respiratory system resistance (R_aw_) was calculated as (21):


$$\begin{array}{l} \;{\text{R}}_{\text{aw}}\;\left(\text{cm}{\text{H}}_{2}\text{O L se}{\text{c}}^{-1}\right)\;=\;\left({\text{P}}_{\text{peak}}\;-\;{\text{P}}_{\text{plat}}\right)\;/\text{ inspiratory flow} \end{array}$$

Where P_peak_ is the airway pressure measured immediately prior to the end-inspiratory pause. Elastance values are reported indexed by kg of IBW. Resistance values are reported indexed by kg of IBW^−0.891^ (22).

The stress index (SI) was estimated using a validated approach based on the Levenberg-Marquardt method (23). Briefly, during constant flow inflation, the dynamic pressure-time curve can be described by a power equation:


$$\begin{array}{l} \;{\text{P}}_{\text{aw}}\;\left(\text{t}\right)\;=\text{ a }\times \;{\text{t}}^{\text{b}}\;+\text{c} \end{array}$$

Where t is time and a, b and c are constant coefficients. The coefficient b (SI) is a dimensionless number that describes the shape of the pressure-time curve. When b < 1, the pressure-time curve presents a downward concavity, indicating that elastance decreases with time during the breath. When b > 1, the pressure-time curve presents an upward concavity, indicating that intratidal elastance increases with time. When b = 1, the pressure-time curve is straight, indicating that elastance is constant over time. In the present study, an SI < 0.95 is defined as tidal recruitment/derecruitment (RD) and an SI > 1.05 is defined as tidal overdistension, whereas a straight line (0.95 > SI < 1.05) is defined as a constant elastance (24). An example obtained from the analysis of one dog is shown in Supplementary Figure 1. This evaluation was performed with an automated software (ICU Lab, KleisTEK Engineering, Italy).

### Statistical Analysis

The Shapiro-Wilk test was used to test data for normality. Data are reported as mean ± SD or median [range] where appropriate. Differences in respiratory mechanics, arterial blood gases and CT scan variables between treatments were evaluated with linear mixed models (for normally distributed data) and with generalized linear mixed models (for non-normally distributed data) with time, treatment, and the interaction between time and treatment as fixed effects, and dog ID and the interaction between dog ID and treatment as random effects. Differences in aeration at end-expiration between regions in the ventrodorsal direction were compared with two-way ANOVA followed by Tukey test for multiple comparisons. Simple linear regression was used to evaluate the association between: (a) body fat percentage and E_RS_, E_L_, and E_CW_; (b) body fat percentage and lung aeration at end-expiration; (c) body fat percentage and FRC indexed by kg of IBW (FRC kg^−1^ IBW); and (d) body fat percentage and R_aw_ indexed by kg of IBW^−0.891^, and the coefficients of determination (*R*^2^) reported. Due to the uneven distribution of the nominal categorical variable “body conformations” and the small number of dogs included, no analysis was performed on the association of this variable with the evaluated outcomes. A *p* < 0.05 was considered significant. All statistical analyses were performed using JMP Pro 14 (SAS, USA) and GraphPad Prism 8 (GraphPad Software, USA).

## Results

### Patient Characteristics and Gas Exchange

Six adult (2–9 years of age) dogs (1 male, 5 female) with a body condition score between 8 and 9 and a percentage of body fat between 20 and 43% were included in the study (Table 1). The arterial partial pressure of O_2_ (PaO_2_, mmHg) was not different (*p* = 0.977) between treatments [536 (232–584) and 514 (280–533) during VTc and VTi, respectively]. No differences were found (*p* = 0.999) in the F-shunt index (%) between VTc (11.5 ± 8.7) and VTi (11.3 ± 6.6) or in the PaCO_2_ (mmHg) (38.8 ± 5.7 and 43.8 ± 8.8 during VTc and VTi, respectively).

**Table 1:** Patient characteristics in a group of six anesthetized obese dogs with varying degrees of body fat percentage undergoing computed tomography scans, and ventilated with tidal volumes based on current vs. ideal body weight.

**Cases**	**Sex**	**Age (months)**	**Current weight (kg)**	**Ideal weight (kg)**	**Body fat percentage (%)**	**BCS**	**Breed**	**Chest conformation**
						**(1–9)**		
1	M	48	45	40.4	20	9	Corso	Deep
2	F	60	15	10.2	33	9	Mixed	Intermediate
3	F	24	12	8.7	27	9	Mixed	Barrel
4	F	72	19	13	32	8	Mixed	Intermediate
5	F	108	17	10	43	9	Mixed	Intermediate
6	F	60	19	12	35	9	Mixed	Deep

*BCS, body condition score; F, female; M, male*.

### CT Scan

#### End-Expiration

**Figure 2:**
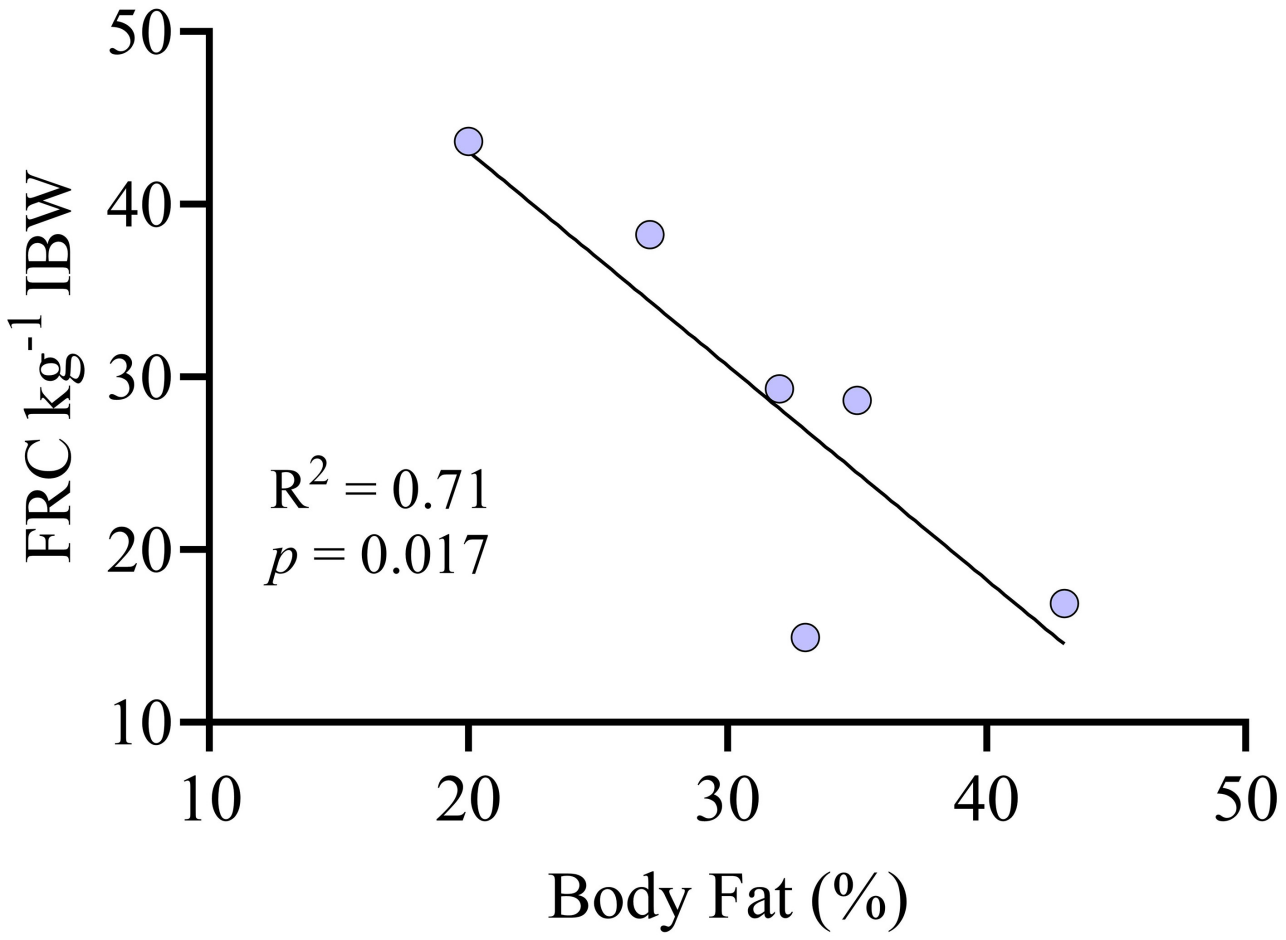
Correlation between the percentage of body fat and the functional residual capacity (FRC) indexed by ideal body weight (kg) in six anesthetized obese dogs undergoing CT scans.

**Figure 3:**
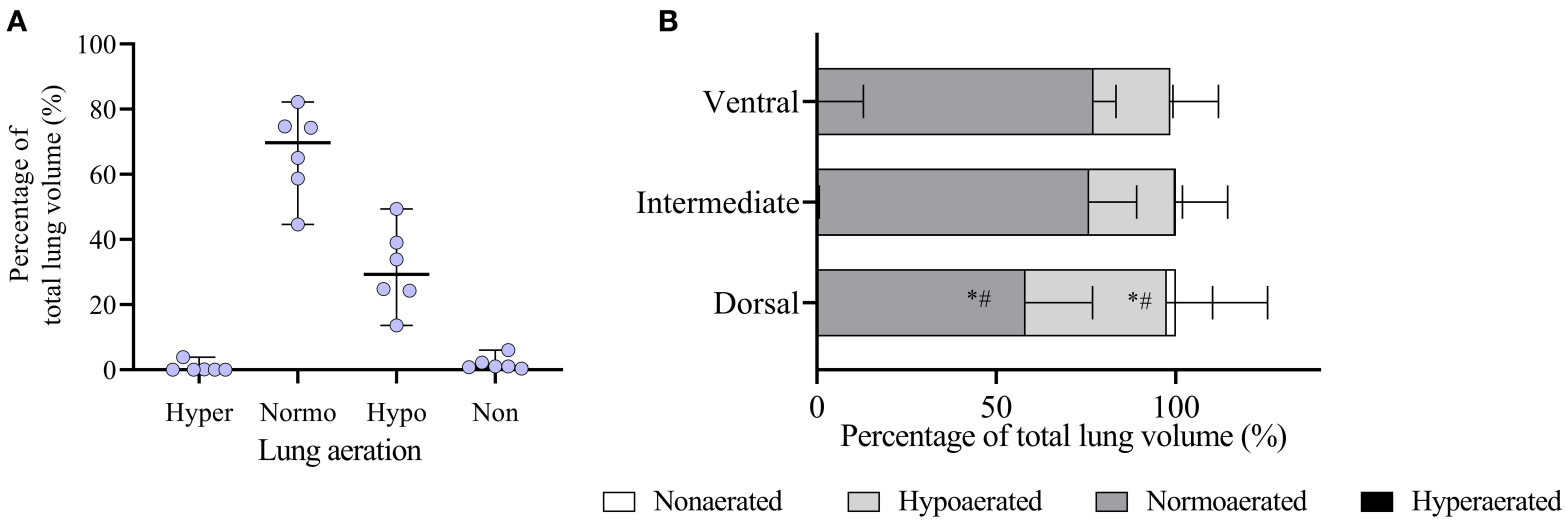
Analysis of lung aeration data obtained by computed tomography (CT) scans during an end-expiratory pause in six obese dogs undergoing anesthesia. **(A)** Whole lung aeration analysis. Hyper, hyperaerated; Normo, normoaerated; Hypo, hypoaerated; Non, nonaerated. **(B)** Regional analysis of aeration in the ventrodorsal direction is shown. Since dogs were in dorsal recumbency, the ventral region represents the nondependent lung and the dorsal region represents the dependent lung. *p < 0.05 compared to normoaeration in the ventral and intermediate regions; ^#^*p* < 0.05 compared to hypoaeration in the ventral and intermediate regions.

The FRC was 28.6 ± 11.4 mL kg^−1^ of IBW. There was a significant negative correlation between FRC kg^−1^ IBW and the percentage of body fat (Figure 2). In whole lung evaluation, percentages of lung volume with hyperaeration (0.03% [0.00–3.35]) and nonaeration (1.06% [0.37–6.02]) were very low (Figure 3A). The percentage of normally aerated and hypoaerated lung volume was 69.7% [44.6–82.2] and 29.3 [13.6–49.4], respectively (Figure 3A). There was a significant (*p* = 0.014) positive correlation between percentage of hypoaeration and body fat percentage (*R*^2^ = 0.82). The percentage of normoaeration showed a nonsignificant (*p* = 0.051) negative correlation (*R*^2^ = 0.65) and the percentage of nonaeration showed a nonsignificant (*p* = 0.099) positive correlation (*R*^2^ = 0.53) with body fat percentage.

**Figure 4:**
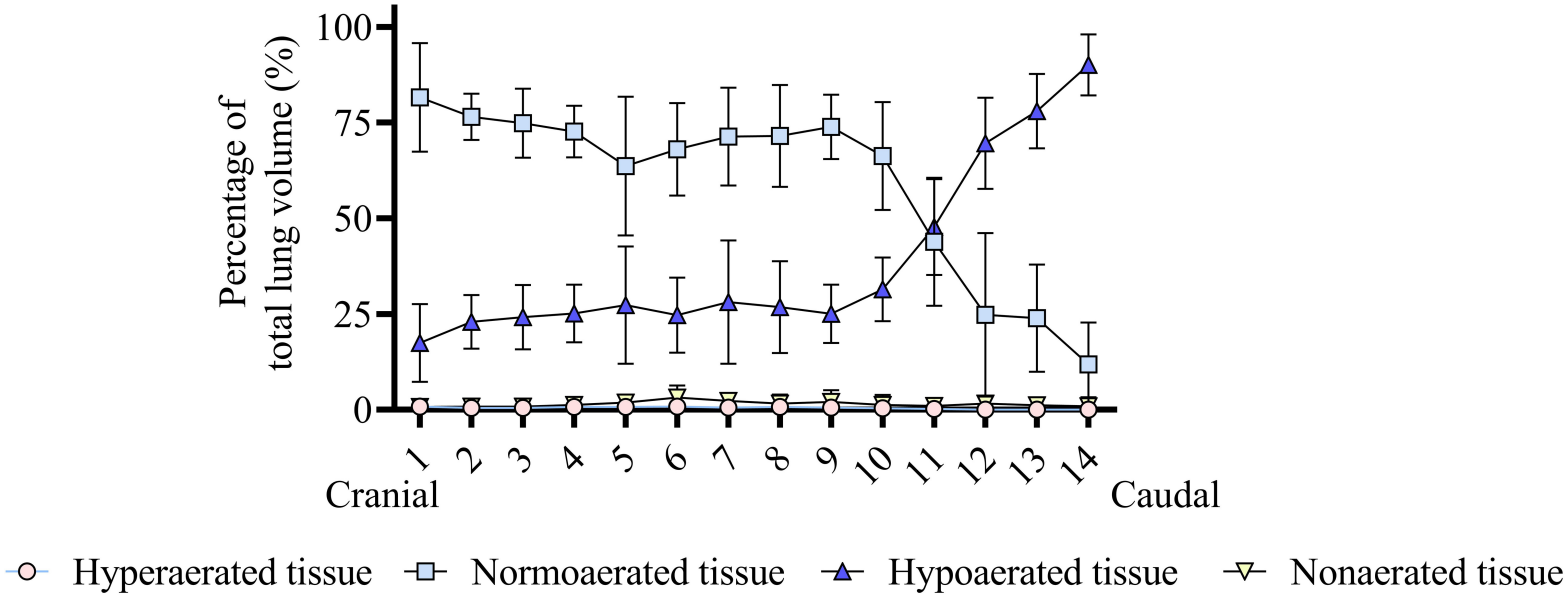
Analysis of the craniocaudal distribution of lung aeration in six obese dogs undergoing anesthesia. It can be seen that most of the midcranial lung tissue is normoaerated, whereas hypoaeration becomes predominant in the caudal lung. Hyperaerated tissue and nonaerated tissue are scarce.

In the ventrodorsal direction, the percentage of hypoaerated tissue was higher (*p* < 0.001) and the percentage of normally aerated tissue was lower (*p* < 0.001) in the dorsal region compared with the ventral and intermediate regions (Figure 3B). Figure 4 shows that in the craniocaudal direction, most of the midcranial lung tissue was normoaerated (~75%), with the remaining being mostly hypoaerated. In the most caudal slices, the percentage of normoaeration decreased and that of hypoaeration increased, reaching 90% of lung tissue in the slice closest to the diaphragm (see Figure 4). Supplementary Videos 1, 2 show the distribution of hypoaerated lung tissue in the reconstructed lungs of a lean (from an ongoing, unpublished study) and an obese dog (from the present study), respectively.

#### End-Inspiration

**Figure 5:**
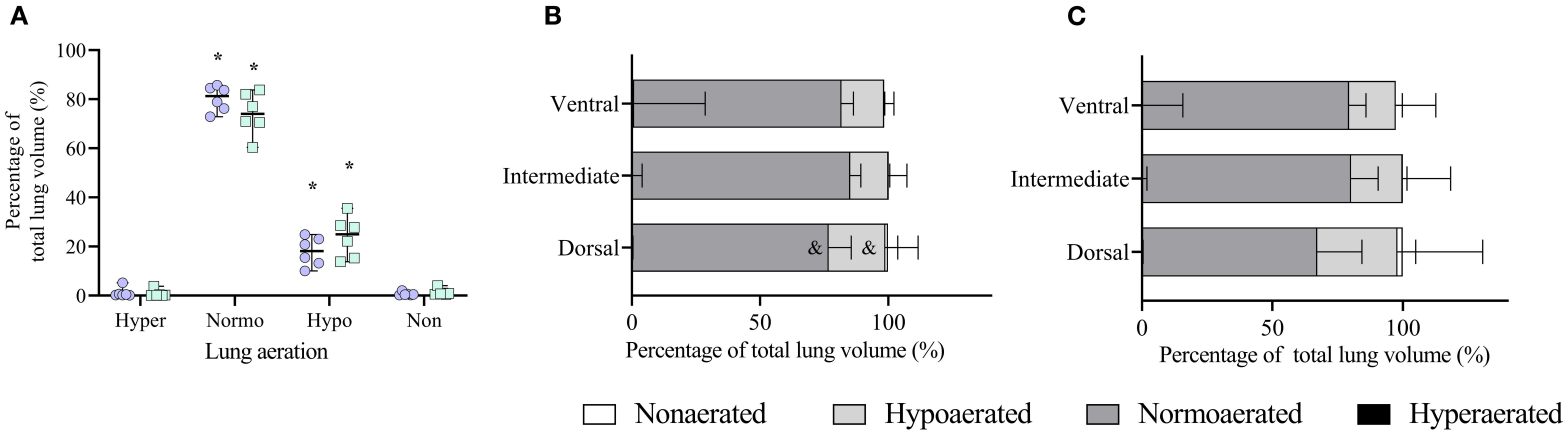
Analysis of lung aeration data obtained by computed tomography (CT) scans during an end-inspiratory pause in six obese dogs undergoing anesthesia. **(A)** Whole end-inspiratory aeration is shown during ventilation with tidal volumes based on current (VTc, open circles) and ideal (VTi, open squares) body weight. Hyper, hyperaerated; Normo, normoaerated; Hypo, hypoaerated; Non, nonaerated. *p < 0.05 compared to the respective aeration during end-expiration (Figure 4A). **(B)** Regional end-inspiratory aeration during treatment VTc.^&^*p* < 0.05 compared to the respective aeration within the same region during end-expiration (Figure 3B). **(C)** Regional end-inspiratory aeration during treatment VTi.

In whole-lung evaluation at end-inspiration, normoaerated tissue increased (*p* = 0.024) and hypoaerated tissue decreased (*p* = 0.027) compared to end-expiration, with both tidal volumes (Figure 5A). There were no differences in aeration between treatments in whole-lung evaluation.

In the ventrodorsal direction during VTc, normally aerated tissue increased (*p* = 0.005) whereas hypoaerated (*p* = 0.007) and nonaerated tissue decreased (*p* < 0.032) only in the dorsal region compared with the end-expiratory images (Figure 5B). There were no differences between end-expiration and end-inspiration during VTi in any of the regions (Figure 5C). No regional differences were seen between VTc and VTi at end-inspiration.

#### Volumetric Lung Strain

**Figure 6:**
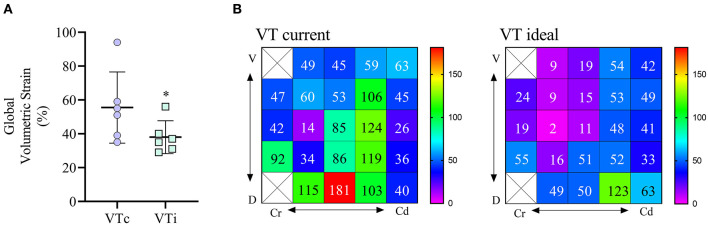
Global and regional volumetric strain calculated for six obese dogs undergoing anesthesia for computed tomography (CT) scan. **(A)** Global volumetric strain in dogs receiving a tidal volume based on current (VTc) and ideal (VTi) body weight. *p < 0.05. **(B)** Regional strain 2D maps are shown for one dog receiving VT based on current body weight (left matrix) and based on ideal body weight (right matrix). Values are percentages, the void squares indicate that no lung intersection between ventrodorsal and craniocaudal directions was found in these ROIs.

As shown in Figure 6A, global lung strain was significantly (*p* = 0.014) higher with VTc compared with VTi. In the individual dog in which regional volumetric strains were calculated, the global volumetric strain during VTc was 54%, whereas during VTi was 34%. However, regional volumetric strains ranged from 14 to 181% during VTc and from 2 to 123% during VTi (Figure 6B). Regional strains were higher during VTc in 20 of 23 regions evaluated in this dog.

### Respiratory System Mechanics

Respiratory mechanics are shown in Table 2. Global mechanics were obtained in all dogs, whereas partitioned mechanics were not properly obtained in two animals, therefore these results are based on four dogs only. Tidal volume was higher (*p* = 0.045) during VTc compared with VTi. Tidal volume indexed by current body weight (mL kg^−1^) was higher (*p* < 0.001) during VTc than during VTi. Peak airway pressure, P_plat_, P_ESplat_, and ΔP_awO_ were significantly higher during VTc compared with VTi (*p* < 0.05). Other variables were not different between treatments. End-expiratory P_TP_ was negative. Respiratory system resistance indexed by IBW^−0.891^ showed a positive correlation with body fat percentage (*R*^2^ = 0.75, *p* = 0.024).

**Table 2:** Global and partitioned respiratory system mechanics in a group of six obese anesthetized dogs undergoing computed tomography scans, and ventilated with tidal volumes based on current vs. ideal body weight.

**Variable**	**V_**T**_ current**	**V_**T**_ ideal**	***p-*value**
VT (mL)	323 ± 75	240 ± 73	<0.001
VT kg^−1^ current (mL kg^−1^)	15.2 ± 0.2	10.7 ± 0.6	0.001
P_peak_ (cmH_2_O)	15.9 ± 1.3	12.2 ± 1	<0.001
P_plat_ (cmH_2_O)	10.2 ± 0.6	7.9 ± 0.4	0.046
PEEP_RS_ (cmH_2_O)	0.3 ± 0.1	0.3 ± 0.1	0.947
P_ESplat_ (cmH_2_O)	6.8 ± 0.5	6.2 ± 0.4	0.046
PEEP_CW_ (cmH_2_O)	2.7 ± 0.9	2.7 ± 0.9	0.671
ΔP_awO_ (cmH_2_O)	9.8 ± 0.7	7.6 ± 0.4	0.002
ΔP_ES_ (cmH_2_O)	3.9 ± 0.3	3.4 ± 0.2	0.061
ΔP_L_ (cmH_2_O)	6.0 ± 1.1	4.1 ± 0.5	0.062
E_RS_ IBW^−1^ (cmH_2_O L^−1^ kg^−1^)	2.6 ± 1.6	2.3 ± 1.5	0.309
E_L_ IBW^−1^ (cmH_2_O L^−1^ kg^−1^)	1.5 ± 1.2	1.2 ± 0.9	0.394
E_CW_ IBW^−1^ (cmH_2_O L^−1^ kg^−1^)	1.0 ± 0.7	1.1 ± 0.7	0.461
E_L_/E_RS_	0.56 ± 0.06	0.52 ± 0.04	0.062
EE P_L_ (cmH_2_O mL^−1^)	−2.3 ± 0.9	−2.3 ± 0.9	>0.999
R_aw_ (cmH_2_O L s^−1^ kg IBW^−0.819^)	2.2 ± 1.6	1.6 ± 1.3	0.157
Respiratory rate (per min)	14 ± 6	22 ± 7	0.130

*VT, tidal volume; VT kg^-1^ current, tidal volume by current body weight; VT kg^-1^ ideal, tidal volume by ideal body weight; Ppeak, peak airway pressure; P_plat_, plateau airway pressure; PEEP_RS_, positive end-expiratory pressure of the respiratory system; P_ESplat_, plateau of the esophageal pressure; PEEP_CW_, positive end-expiratory pressure of the chest wall; ΔP_awO_, driving airway pressure; ΔP_ES_, driving esophageal pressure; ΔP_L_, driving transpulmonary pressure; E_RS_ elastance of the respiratory system; E_L_ elastance of the lung; E_CW_, elastance of the chest wall; E_L_/E_RS_, ratio of the elastance of the lung to the elastance of the respiratory system; EE P_L_, end-expiratory transpulmonary pressure; R_aw_ respiratory system resistance indexed by ideal body weight^−0.891^. Variables requiring the value of esophageal pressure for its calculation (P_ESplat_, PEEP_CW_, ΔP_ES_, ΔP_L_, E_L_, E_CW_, E_L_/E_RS_, and EE P_L_) were obtained from four dogs. All other variables were calculated from six dogs*.

**Figure 7:**
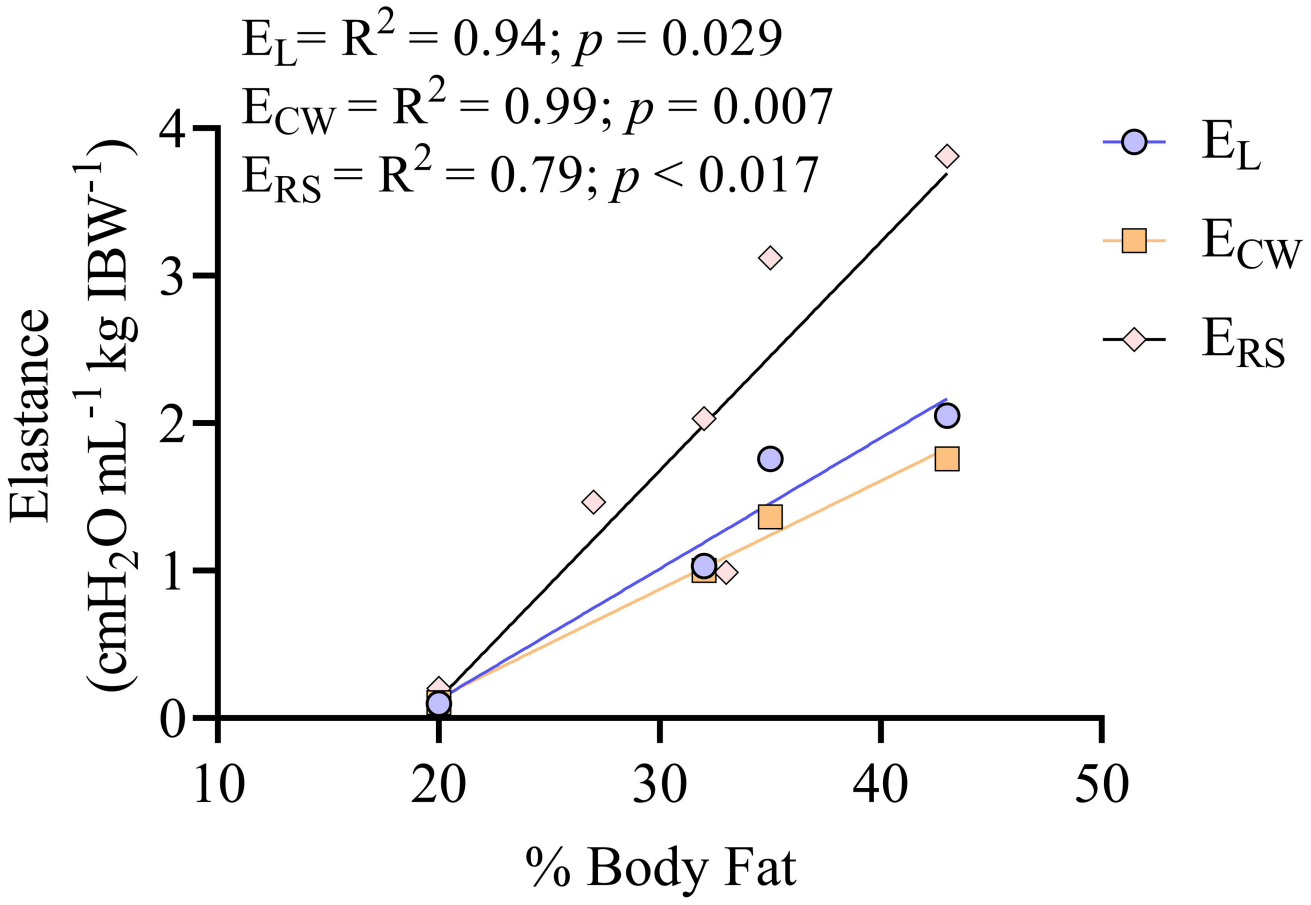
Correlation between percentage (%) of body fat and the different elastances of the respiratory system in six obese dogs undergoing anesthesia. There are only four dogs for the elastance of the lung (E_L_) and elastance of the chest wall (E_CW_), since two dogs had no readings of esophageal pressure. Six dogs are shown for the correlation between the percentage of body fat and the elastance of the respiratory system (E_RS_).

**Figure 8:**
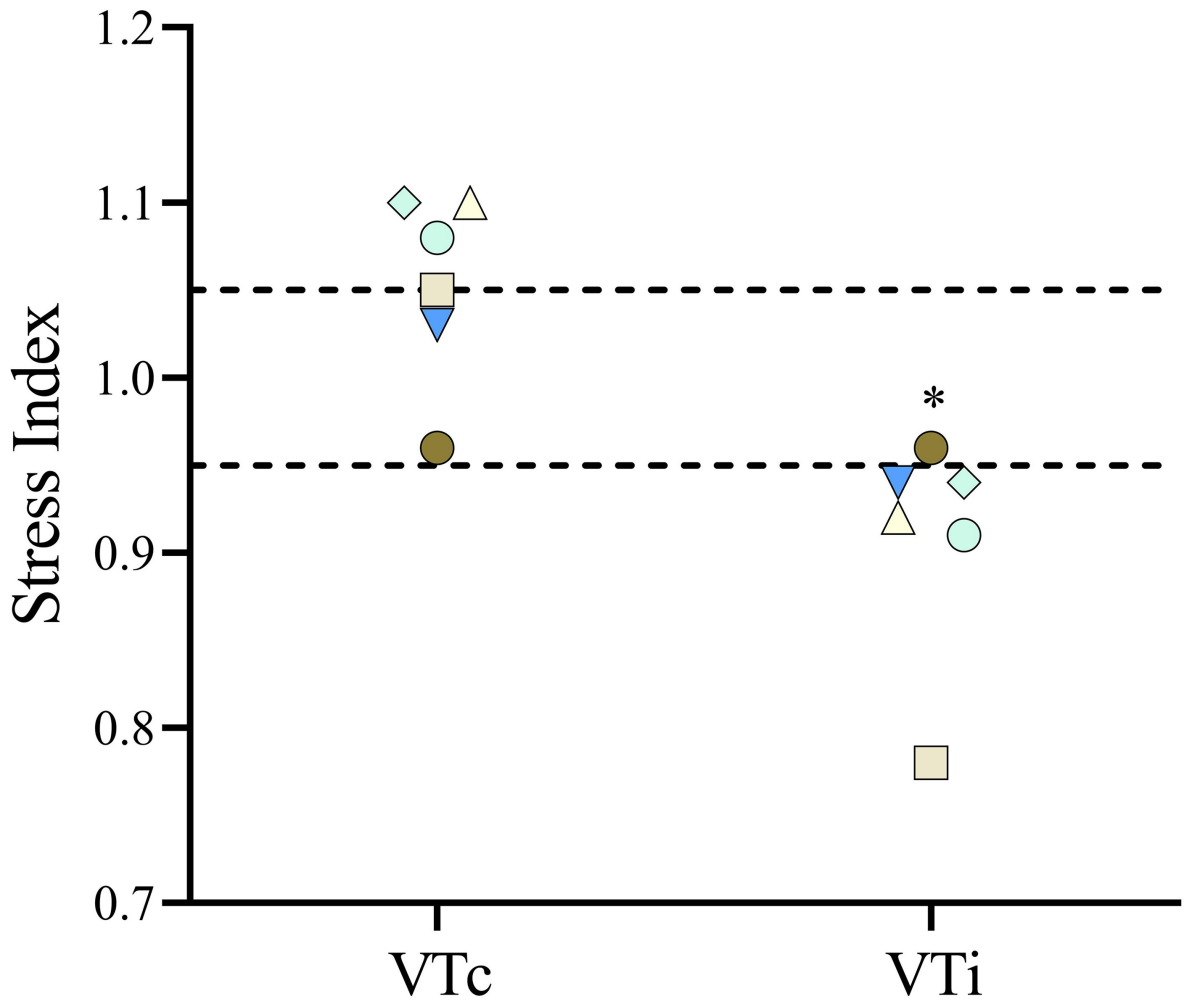
The stress index was calculated in six obese dogs undergoing anesthesia receiving a tidal volume based on current (VTc) and ideal (VTi) body weight. In the figure, each dog is represented by the same symbol and color during both tidal volumes. *p = 0.012.

The percentage of body fat showed a positive correlation with E_RS_, E_L_, and E_CW_, as shown in Figure 7. The SI (Figure 8) was higher (*p* = 0.012) during VTc (SI = 1.07 [0.14]) compared with VTi (SI = 0.93 [0.18]).

## Discussion

This study represents an initial characterization of partitioned respiratory system mechanics and thoracic CT findings in obese dogs undergoing general anesthesia and mechanical ventilation with VT based on ideal vs. current body weight. The results of the present work can be summarized as follows. First, in this small cohort of obese adult dogs, there is a high percentage of hypoaerated tissue, especially in the most dependent areas and those close to the diaphragm. Interestingly, the percentage of hypoaeration increases linearly with the percentage of body fat. Second, global lung strain and ΔP_awO_ are significantly higher during VTc, whereas global lung strain with VTi is similar to that described for lean dogs (19), suggesting a more physiologic tidal inflation during the latter. Third, although P_plat_ is higher during VTc, elastances are not significantly different compared with VTi. Likewise, R_aw_, indexed by an allometric coefficient, is comparable between treatments. Both the elastances and the R_aw_ are significantly correlated with body fat percentage. Finally, SI, a variable defining the shape of the pressure-time curve during constant flow, suggests tidal overdistension during VTc and tidal RD during VTi.

The prevalence of overweight and obesity in dogs is increasing and some studies report that when included together, up to 45% of the overall dog population may fall into this category (1, 5). Besides inducing changes in several other systems, obesity can significantly alter the normal function of the respiratory system (5). In humans, the accumulation of adipose tissue in the abdomen results in compression of the thorax with ensuing reductions in FRC (2). In the present study, the different elastances normalized to IBW where higher than those previously reported in populations of lean adult anesthetized dogs (19, 25). In ventilated human patients, obesity is associated with increased E_RS_, explained mostly by an increase in the E_L_, and to a lesser extent by the E_CW_. Lung collapse, with reductions in FRC, seem to be responsible for increases in E_L_ and E_RS_ (2, 26). Obese dogs had a large proportion of hypoaerated lung tissue, which is probably responsible for the increased E_L_ and E_RS_ seen in this study. Further studies should evaluate whether these differences are still apparent when comparing respiratory mechanics of lean and obese dogs with similar body conformations. Interestingly, our study also showed a strong correlation between the normalized elastances and the body fat percentage, suggesting that overweight and obesity in anesthetized dogs can lead to significant alterations in respiratory system elastance.

A common finding in obese people is an increased R_aw_, most likely due to reductions in lung volumes (2, 26, 27). Although atelectasis in the present study was very low, as evidenced by the minimal amount of nonaerated lung tissue, hypoaeration was significant and the FRC kg^−1^ IBW was lower compared with a population of lean dogs previously reported by our group (mean FRC kg^−1^ = 37 mL kg^−1^) (19). Moreover, we observed a significant negative correlation between FRC kg^−1^ IBW and body fat percentage that is worth exploring further. Decreased FRC has been ascribed as the main factor behind an increased R_aw_ in obese people. The extent of hypoaerated tissue in the lungs of the dogs in the present study, especially in the ventral and caudal areas, was large. In anesthetized human patients in supine position, most atelectasis has been reported to occur near the diaphragm, and less toward the apex, similar to our results (28). In lean healthy dogs under anesthesia, a similar pattern of distribution has been observed, although the magnitude of hypoaerated tissue is significantly less than that of this population of obese dogs (unpublished data). Supplementary Videos 1, 2 show the 3D distribution of hypoaerated tissue in a lean dog from an unrelated ongoing study and an obese dog from the present study, evidencing significantly more hypoaerated tissue in the obese dog under similar anesthetic conditions and positioning. Interestingly, our results also showed a positive correlation between body fat percentage and R_aw_ kg^−1^ IBW^−0.891^. Therefore, when taken together with the correlation observed between the elastance and body fat percentage, our results suggest that obesity in dogs may increase both the elastic and resistive work of breathing. This could represent a clinical problem, especially for spontaneously breathing obese dogs under sedation or anesthesia.

Aggressive mechanical ventilation leading to excessive parenchymal deformation in people has been associated with a high incidence of postoperative pulmonary complications (29, 30). Therefore, the appropriate setting of intraoperative ventilatory variables seems to play an important role in protecting the lungs from ventilator-induced lung injury. Volumetric lung strain is a measure of cyclic lung deformation and has been found to be one of the main determinants of ventilator-induced lung injury (31–33). In the present study, global volumetric lung strains were significantly lower during VTi compared with VTc. In fact, strains during VTi were comparable to a cohort of lean dogs (19), suggesting that in obese dogs, a protocol using VTi is more physiologic than VTc. The evaluation of regional lung strains in one dog revealed a wide range of values, suggesting that the determination of lung strain using global estimations can over, and more importantly, under estimate local strain values in this population. This is relevant considering that, especially during ventilation with VTc, potentially injurious values of strain developed in some regions (31, 34). Further studies are required to confirm these preliminary findings. Airway driving pressure has been used as a surrogate of global lung strain (33). As expected, VTc generated significantly higher ΔP_awO_ compared with VTi. Other estimates of lung distension such as the ΔP_L_ and the E_L_ were not different between treatments; however, there was a trend to higher values during VTc, which in view of other significant differences, such as ΔP_awO_ and lung strain, suggest that these trends may become significant if the sample size is increased, although the latter remains speculative. Similarly, the mean SI during VTc was at the high-end of the normal range, with two dogs exceeding this cut-off point (24). Although higher SI values suggest the presence of intratidal overdistension, the percentage of hyperaerated tissue in the end-inspiratory images was very low, even during VTc. However, prolonged inspiratory breath holds applied during static end-inspiratory CT imaging of the thorax can lead to significant intratidal redistribution of lung volumes (35), which can render different information compared with a dynamic parameter such as the SI. Future studies should evaluate tidal hyperaeration in obese dogs using such techniques as dynamic CT. It has to be considered, however, that normal SI values for dogs have not been validated. More importantly, prospective studies in obese dogs should evaluate whether protocols using VTi result in lung protection compared to those using VTc. In obese mice, ventilation based on VTc as compared to VTi increases the expression of several pulmonary inflammatory markers (4).

To the authors' best knowledge, this is the first study to report the end-expiratory P_L_ in obese dogs. In people, increased abdominal load leads to the regional generation of positive pleural pressures, which in turn can lead to the generation of negative end-expiratory P_L_, especially in the absence of externally applied PEEP (26). Therefore, some authors advocate titrating PEEP to achieve values of end-expiratory P_L_ that are slightly positive (e.g., +2 cmH_2_O) (26). This negative end-expiratory P_L_ values could in part explain the reduced FRC kg^−1^ and the large proportion of hypoaerated tissue observed in this study. Given the well-described limitations of using absolute esophageal pressure values and calculations derived from it (e.g., end-expiratory transpulmonary pressure), these results should be interpreted cautiously (26, 36). Interestingly, during VTi, the mean SI was 0.93, suggesting that external PEEP in these dogs could have been beneficial to prevent tidal RD and to improve lung aeration (24). Together, these data suggest that PEEP should be titrated in these patients to avoid negative values of end-expiratory P_L_ and to increase lung volumes, regardless of how the VT is set, and to normalize indices of tidal RD such as low SI values during ventilatory protocols using VTi.

An unexpected finding in the present study was the absence of major oxygenation impairment. Obese human patients undergoing mechanical ventilation have significant gas exchange abnormalities, resulting in venous admixture and shunt, associated most likely with the development of extensive atelectasis (2). In obese sedated dogs, oxygenation was impaired and oxygen tension-based indices improved after weight loss, suggesting a similar effect of obesity on oxygenation as that reported for obese humans (7). The reason for the lack of significant oxygenation impairment in the present study is unclear, but it may be related to several factors. For instance, dogs in the present study, unlike sedated obese dogs reported by Mosing et al. (7), were undergoing mechanical ventilation, which could have resulted in improved overall gas exchange and resulted in better oxygenation. Also, an important difference between dogs of this study and that of the sedated dogs is that the percentage of body fat was significantly lower (mean body fat percentage was 30% in the present study vs. 45% in the sedated dogs) (7). Similarly, although in the dogs reported here there was a significant proportion of hypoaerated lung tissue, especially in dependent and caudal areas of the lung, the amount of nonaerated tissue was very low, which could have resulted in scarce intrapulmonary shunt formation and instead, the development of a larger proportion of low ventilation-to-perfusion units. Although areas of low ventilation-to-perfusion ratios can significantly alter gas exchange, these units tend to respond to increased oxygen concentrations by increasing their end-capillary oxygen content (37). Although high fractions of oxygen may lead to absorption atelectasis in some situations (38), recent studies in anesthetized dogs have shown that the effect of this phenomenon on gas exchange may not be as relevant as previously thought in dogs (39, 40). However, further prospective studies should evaluate the effects of extreme obesity with higher percentages of body fat on oxygenation in dogs, as in these cases it is possible that the extent of nonaerated tissue and intrapulmonary shunt may be higher, as is described in morbidly obese people.

Our study has significant limitations. First, the number of animals included is small. This was due to the difficulty in recruiting obese dogs that required anesthesia and mechanical ventilation for diagnostic CT scans. This limitation is even more significant when considering the partitioned respiratory mechanics, as only four dogs had correct measurements of esophageal pressure. Moreover, we used absolute values of esophageal pressure as a surrogate of pleural pressure. Although this approach is methodologically convenient, studies have shown that absolute esophageal pressure measurements may not accurately reflect pleural pressure values in all conditions or areas of the chest (36), although other studies show that, following proper calibration, absolute esophageal pressure measurements are a good estimate of dependent pleural pressures (41). Therefore, future studies in clinical dogs should focus on the impact of these situations in the estimation of pleural pressure. Second, we only focused on the effects of VT on the measured outcomes; however, an equally important consideration regarding the ideal ventilatory settings in these patients is the use of PEEP, especially considering the negative values of end-expiratory P_L_ observed in the present study. In fact, lung strain, an important outcome measured in this study, depends not only on the cyclic variation of tidal volume but also on the baseline end-expiratory volume (i.e., the FRC), largely dependent on PEEP (33). However, since we anticipated a low rate of patient recruitment, our decision to focus only in one variable was pragmatic, in an attempt to capture the effect of the VT *per se*. Another limitation is that we did not include obese dogs undergoing surgery, especially for abdominal procedures and laparoscopies. In these patients, there could be an additive negative effect between obesity and abdominal manipulation or insufflation on the respiratory system (26). We performed only a single end-expiratory evaluation of CT images and respiratory mechanics in each dog, which compounds to the fact that no standardization of lung volume history was performed. This may be an important limitation of the present study and should be kept in mind when interpreting the results. Although, time could have created differences in end-expiratory aeration and mechanics, a study showed that end-expiratory lung volume did not change when ventilating anesthetized dogs with a tidal volume of 8 or 15 mL kg^−1^ (15). Moreover, we decided to omit one end-expiratory measurement in an attempt to reduce experimental duration and to spare dogs from another set of CT images.

In conclusion, in mechanically ventilated obese dogs, as the percentage of body fat increases, FRC decreases while the percentage of pulmonary hypoaeration, R_aw_, E_RS_, E_L_, and E_CW_ increase. Although no difference in gas exchange was noted, mechanical ventilation of obese dogs using VTi generated more physiologic volumetric lung strains and lower ΔP_awO_ than VTc, suggesting that the VTi may be more appropriate than VTc for mechanical ventilation of this canine patient population. Future studies should evaluate the impact of mechanical ventilation with VTi on the clinical outcomes of anesthetized obese dogs.

## Data Availability Statement

The raw data supporting the conclusions of this article will be made available by the authors, without undue reservation.

## Ethics Statement

The animal study was reviewed and approved by Ethical Committee for Clinical Study in Animal Patients of the Department of Emergency and Organ Transplantation, University of Bari, Italy. Written informed consent was obtained from the owners for the participation of their animals in this study.

## Author Contributions

JA: interpreted the data, analyzed the respiratory mechanics and the CT scans, performed the statistics, and wrote the manuscript. LL, VM, MS, and AC: participated in study design and experimental procedure. SG and MM-F: interpreted the data and critically reviewed the manuscript. IP: analyzed the CT scans and created the 3d reconstructions of the lungs. DH and AP: critically reviewed the manuscript and performed the biomechanical analysis of regional strain. FS: designed the study, performed experimental procedures, data analysis, and manuscript review. All authors contributed to the critical revision of the paper and approved the final manuscript.

## Conflict of Interest

The authors declare that the research was conducted in the absence of any commercial or financial relationships that could be construed as a potential conflict of interest.

## Publisher's Note

All claims expressed in this article are solely those of the authors and do not necessarily represent those of their affiliated organizations, or those of the publisher, the editors and the reviewers. Any product that may be evaluated in this article, or claim that may be made by its manufacturer, is not guaranteed or endorsed by the publisher.
