# Supplementary material for: Evaluation of Lung Aeration and Respiratory System Mechanics in Obese Dogs Ventilated With Tidal Volumes Based on Ideal vs. Current Body Weight
Source: Front Vet Sci. 2021 Oct 1;8:704863. doi: 10.3389/fvets.2021.704863 (PMC8517180; doi:10.3389/fvets.2021.704863)
Supplement: Supplementary file 3 [file Data_Sheet_1.docx]

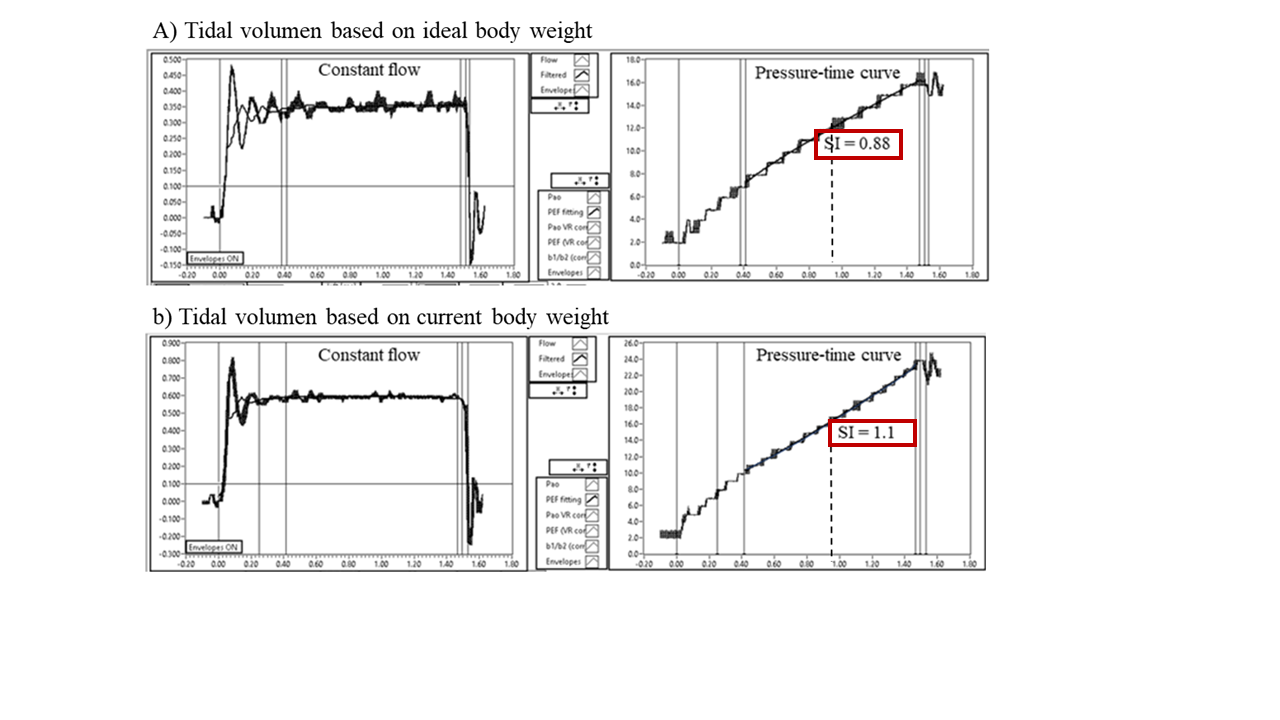


**Supplementary Figure S1**. Example of a stress index (SI) obtained in an obese dog receiving ventilation with tidal volumes based on ideal body weight (A) and current body weight (B). An SI of 1 indicates that airway pressure increases linearly with constant airflow, suggesting that compliance of the respiratory system remains constant during tidal inflation. A SI < 1 indicates intra-tidal recruitment and occurs because the compliance increases as tidal inflation occurs. This is recognized as a pressure-time curve with a downward concavity (Fig 1A). A SI > 1 represents tidal overdistension, and occurs because the compliance decreases as tidal inflation occurs, manifested as a pressure-time curve with an upward concavity.
